# Natural vectors of *Plasmodium knowlesi* and other primate, avian and ungulate malaria parasites in Narathiwat Province, Southern Thailand

**DOI:** 10.1038/s41598-023-36017-3

**Published:** 2023-06-01

**Authors:** Surasuk Yanmanee, Sunee Seethamchai, Napaporn Kuamsab, Sunate Karaphan, Wannapa Suwonkerd, Somchai Jongwutiwes, Chaturong Putaporntip

**Affiliations:** 1 Molecular Biology of Malaria and Opportunistic Parasites Research Unit, Department of Parasitology, Faculty of Medicine, Chulalongkorn University, Bangkok, Thailand; 2 Department of Biology, Faculty of Science, Naresuan University, Pitsanulok, Thailand; 3 Community Public Health Program, Faculty of Health Science and Technology, Southern College of Technology, Nakhon Si Thammarat, Thailand; 4 Department of National Parks, Wildlife and Plant Conservation, Ministry of National Resources and Environment, Bangkok, Thailand; 5 Division of Vector Borne Diseases, Department of Disease Control, Ministry of Public Health, Nonthaburi, Thailand

**Keywords:** Ecology, Microbiology, Molecular biology, Medical research

## Abstract

To date, four species of simian malaria parasites including *Plasmodium knowlesi*, *P. cynomolgi*, *P. inui* and *P. fieldi* have been incriminated in human infections in Thailand. Although the prevalence of malaria in macaque natural hosts has been investigated, their vectors remain unknown in this country. Herein, we performed a survey of *Anopheles* mosquitoes during rainy and dry seasons in Narathiwat Province, Southern Thailand. Altogether 367 *Anopheles* mosquitoes were captured for 40 nights during 18:00 to 06:00 h by using human-landing catches. Based on morphological and molecular identification, species composition comprised *An. maculatus* (37.06%), *An. barbirostris* s.l. (31.34%), *An. latens* (17.71%), *An. introlatus* (10.08%) and others (3.81%) including *An. umbrosus* s.l., *An. minimus*, *An. hyrcanus* s.l., *An. aconitus, An. macarthuri* and *An.* *kochi*. Analyses of individual mosquitoes by PCR, sequencing and phylogenetic inference of the mitochondrial cytochrome genes of both malaria parasites and mosquitoes have revealed that the salivary gland samples of *An. latens* harbored *P. knowlesi* (n = 1), *P. inui* (n = 2), *P. fieldi* (n = 1), *P. coatneyi* (n = 1), *P. hylobati* (n = 1) and an unnamed *Plasmodium* species known to infect both long-tailed and pig-tailed macaques (n = 2). The salivary glands of *An. introlatus* possessed *P. cynomolgi* (n = 1), *P. inui* (n = 1), *P. hylobati* (n = 1) and coexistence of *P. knowlesi* and *P. inui* (n = 1). An avian malaria parasite *P. juxtanucleare* has been identified in the salivary gland sample of *An. latens*. Three other distinct lineages of *Plasmodium* with phylogenetic affinity to avian malaria species were detected in *An. latens*, *An. introlatus* and *An. macarthuri*. Interestingly, the salivary gland sample of *An. maculatus* contained *P. caprae*, an ungulate malaria parasite known to infect domestic goats. Most infected mosquitoes harbored multiclonal *Plasmodium* infections. All *Plasmodium*-infected mosquitoes were captured during the first quarter of the night and predominantly occurred during rainy season. Since simian malaria in humans has a wide geographic distribution in Thailand, further studies in other endemic areas of the country are mandatory for understanding transmission and prevention of zoonotic malaria.

## Introduction

*Plasmodium knowlesi* is endemic in Southeast Asia where natural reservoir hosts including long-tailed (*Macaca fascicularis*) and pig-tailed (*M. nemestrina*) macaques are ubiquitous^1^. The distribution of malaria caused by *P. knowlesi* seems to coincide with the habitats of macaque natural hosts in this region^2,3^. Despite differential prevalence of human infections with *P. knowlesi* across these endemic countries, ranging from relatively low to high infection rates among indigenous people, it is considered to be a virulent malaria species implicating in severe and fatal illnesses in patients with high parasitemia^4,5^. From a global perspective, the number of *P. knowlesi*-infected individuals may be incomparable to those caused by *P. falciparum* and *P. vivax*^6^. However, this simian malaria species poses an important health problem for people living in high transmission areas as well as travelers to these endemic countries^7,8^. It is noteworthy that these macaque natural hosts have a wide geographic distribution where humans and macaques interface in several locations in Southeast Asia. Therefore, the zoonotic nature of knowlesi malaria makes it recalcitrant to control by conventional strategies deployed for human malaria. Besides *P. knowlesi*, other simian malaria species including *P. cynomolgi*, *P. inui*, *P. fieldi* and possible other non-human *Plasmodium* species are implicated in human infections albeit with relatively lower prevalence^8–14^. Therefore, vector control could be an alternative measure for prevention, reduction or elimination of simian malaria transmissible to humans.

Attempts to identify mosquito vectors of *P. knowlesi* and other simian *Plasmodium* species circulating in South and Southeast Asia over the past seven decades were entirely based on experimental transmission of primate malaria parasites between laboratory-rear mosquitoes and monkeys^1^. For *P. knowlesi*, it has been demonstrated that complete sporogonic development of this parasite was achieved in at least 12 species of *Anopheles* consisting of *An. annularis*, *An. aztecus*, *An. stephensi*, *An. atroparvus*, *An. balabacensis balabacensis*, *An. freeborni*, *An. maculatus, An. vagas*, *An. introlatus*, *An.* *kochi*, *An. sinensis* and *An. quadrimaculatus*^1^. However, some of these mosquitoes have not been identified in areas where knowlesi malaria is endemic. After the first discovery of naturally acquired symptomatic human infection of *P. knowlesi* in 1965^15^, followed by almost three decades later for subsequent identification of this simian malaria in humans in Southeast Asia, a number of *Anopheles* species belonging to the Leucosphyrus Group have been reported to harbor *P. knowlesi* sporozoites or its DNA in their salivary glands^16^. These include *An. hackeri*^17^, *An. cracens*^18^, *An. latens*^19,20^, *An. introlatus*^21^, *An. balabacensis*^22–26^, *An. dirus*^27,28^. Furthermore, *P. knowlesi* has been identified in other groups of *Anopheles* mosquitoes including *An. collessi, An. roperi* and *An. donaldi* in Malaysian Borneo^23,25,29^, and *An. sundaicus* in Andaman and Nicobar Islands^30^. Meanwhile, several vectors of *P. knowlesi* may also have potential to transmit other simian malaria parasites in which detailed information about these mosquitoes has been comprehensively reviewed by Vythilingam et al.^16^.

In Thailand, at least four species of simian *Plasmodium* species have been incriminated in symptomatic infections in humans which included *P. knowlesi*, *P. cynomolgi*, *P. inui* and *P. fieldi*^8,10–13^. Simian malaria in humans has a wide geographic distribution in Thailand albeit differential prevalence of infections occurred across endemic areas with relatively more cases detected in the southern part of the country where macaque natural hosts are more abundant than other regions^10–13,31,32^. To date, it remains unknown whether these simian malaria parasites in Thailand are transmitted by the same mosquito species as those reported from other endemic countries. Since the bionomics of each *Anopheles* species may differ, it is important to identify natural vectors of these simian malaria parasites in each endemic area. Therefore, we performed a prospective investigation of *Anopheles* potential vectors of simian malaria in Southern Thailand. Surveys of *Anopheles* mosquitoes in Narathiwat Province have revealed potential vectors of at least six known species of nonhuman primate malaria parasites as well as four avian and an ungulate *Plasmodium* species. These findings have provided baseline entomological data for simian malaria in Thailand that are important for prevention and control policy.

## Results

### Distribution and abundance of *Anopheles* species

**Figure 1:**
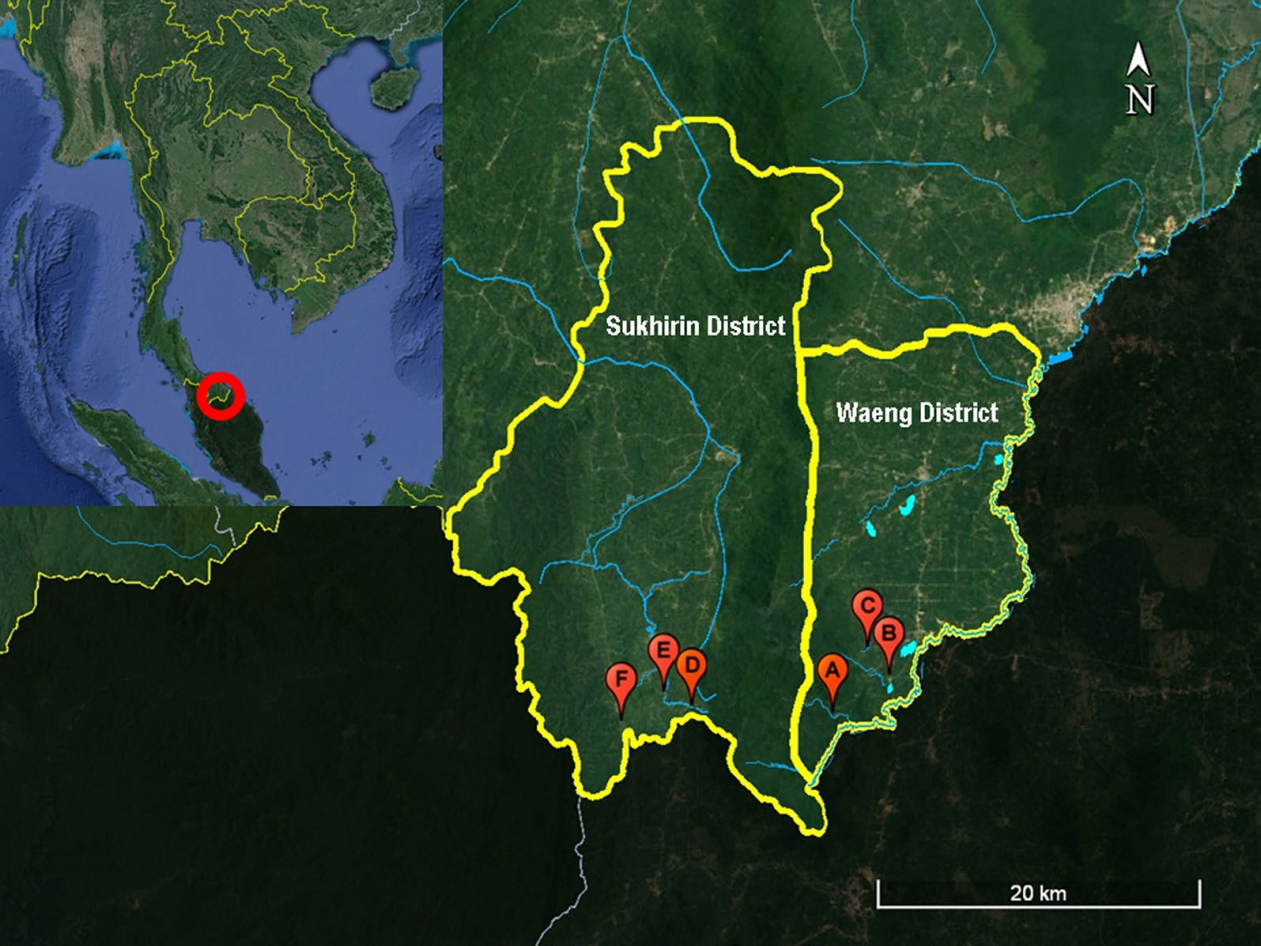
Locations of mosquito collection sites in Waeng (**A–C**) and Sukhirin (**D–F**) Districts, Narathiwat Province, Thailand. The images were from Google Earth Pro 7.3.6.9345 (https://www.google.com/intl/en_uk/earth/versions/#earth-pro)^65^.

**Figure 2:**
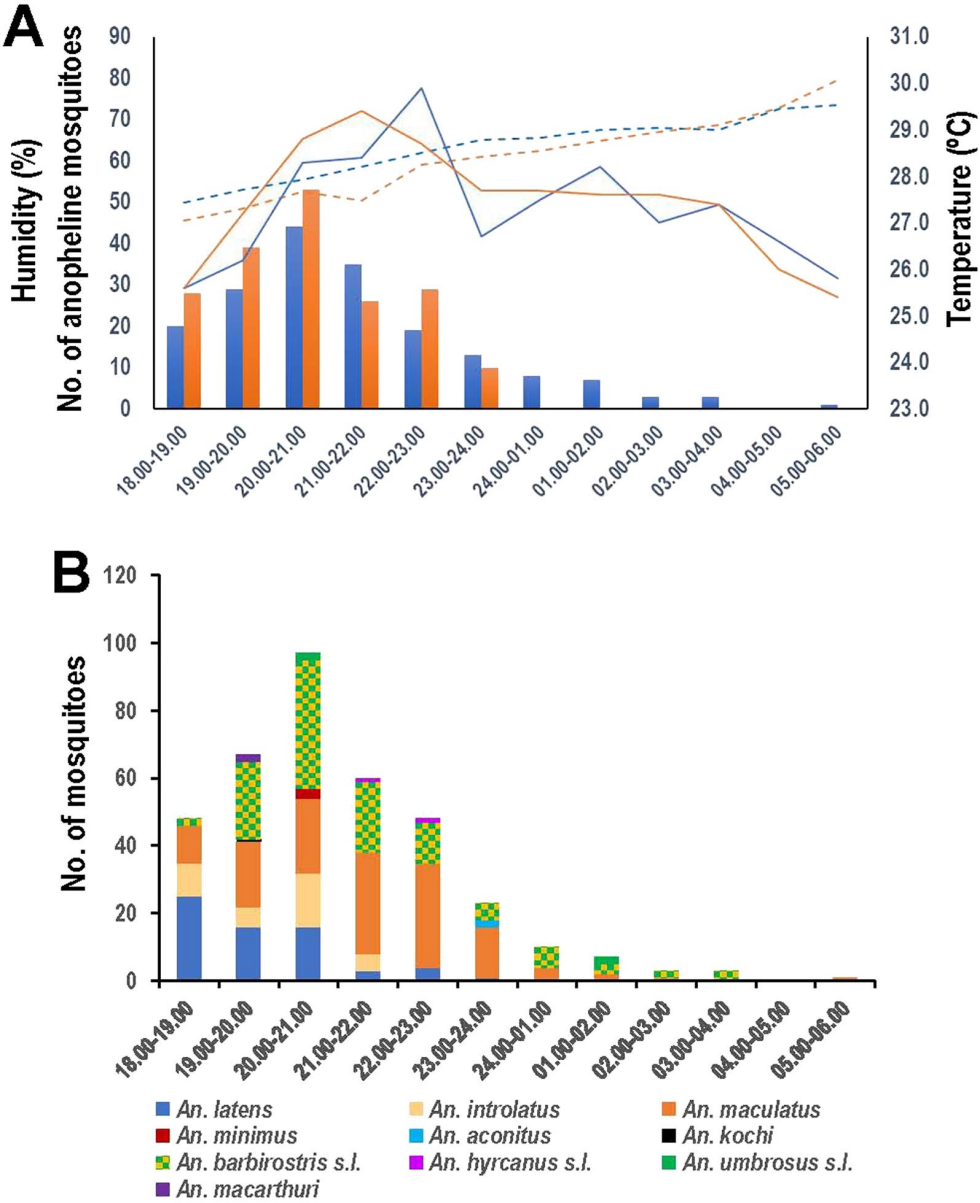
Human-biting patterns of *Anopheles* mosquitoes in Narathiwat Province. (**A**) Total number of mosquitoes collected per hour in 2018 (blue bars) and 2019 (orange bar) in relation to hourly changes in temperature and humidity shown in corresponding colors. (**B**) Abundance of *Anopheles* species/group collected per hour.

Altogether, 367 female *Anopheles* mosquitoes were caught during rainy and dry seasons in 2018 and 2019. The total number of *Anopheles* collected from Sukhirin and Waeng Districts in Narathiwat Province during the raining seasons was 2.7 times more than those captured during the dry seasons (Fig. 1, Table 1). Based on taxonomic keys and molecular analysis of *Anopheles*^33^, the predominant species belonged to *An. maculatus*, *An. barbirostris* s.l., *An. latens* and *An. introlatus* accounting for 96.19% of all *Anopheles* mosquitoes while those identified as *An. umbrosus* s.l., *An. hyrcanus* s.l., *An. minimus*, *An. aconitus* and *An.* *kochi* were sporadically found, ranging from 1 to 4 specimens for each species (Table 1). The biting rates for *An. maculatus*, *An. barbirostris* s.l., *An. latens* and *An. introlatus* were 3.400, 2.875, 1.625 and 0.925 mosquito collected/night/collector, respectively. The number of *Anopheles* mosquitoes captured for each corresponding period of the night between 2018 and 2019 seemed to show a similar trend. The overall number of mosquito collection peaked at 20:00 and 21:00 h whereas remarkably fewer numbers of mosquitoes could be caught after midnight, especially after 2:00 h until daybreak (Fig. 2A). In the mosquito collection sites, the level of temperature peaked during 21:00 and 23:00 h whereas humidity gradually rose from dusk till dawn. The abundance of mosquitoes during the first quarter of the night (18:00 and 21:00 h) seemed to be positively correlated with the environmental temperature (Pearson *r* = 0.999, *p* = 0.03) and roughly correlated with the levels of humidity (Pearson *r* = 0.996, *p* = 0.05) (Fig. 2A). The number of *An. latens* and *An. introlatus* seemed to be early feeders from 18:00 and 19:00 h whereas peak feeding time of *An. barbirostris* s.l. was between 20:00 and 21:00 h. Meanwhile, *An. maculatus* was most abundant from 21:00 to 23:00 h. Despite the decline in the number of mosquitoes after midnight, both *An. maculatus* and *An. barbirostris* s.l. could be sparsely caught before dawn (Fig. 2B).

**Table 1:** Distribution of *Anopheles* in Sukhirin and Waeng Districts, Narathiwat Province.

*Anopheles* group or species*	Dry season** n (%)	Wet season^#^ n (%)	Total n (%)	Biting rate^§^
*An. maculatus*	56 (56.57)	80 (29.85)	136 (37.06)	3.400
*An. barbirostris* s.l.	30 (30.30)	85 (31.72)	115 (31.34)	2.875
*An. latens*	5 (5.05)	60 (22.38)	65 (17.71)	1.625
*An. introlatus*	5 (5.05)	32 (11.94)	37 (10.08)	0.925
*An. umbrosus* s.l.	2 (2.02)	2 (0.75)	4 (1.09)	0.100
*An. minimus*	0 (0)	3 (1.12)	3 (0.82)	0.075
*An. macarthuri*	0 (0)	2 (0.75)	2 (0.54)	0.050
*An. aconitus*	0 (0)	2 (0.75)	2 (0.54)	0.050
*An. hyrcanus* s.l.	1 (1.01)	1 (0.37)	2 (0.54)	0.050
*An.kochi*	0 (0)	1 (0.37)	1 (0.27)	0.025
Total	99 (26.98)	268 (73.02)	367 (100)	–

*Morphological identification was based on *Anopheles* taxonomic keys^33^. Mosquitoes in the Leucosphyrus Group were identified by either species-specific PCR assay^62^ or phylogenetic inference of the *cox1* sequences.

**March 2018 and March 2019.

^#^August 2018 and November 2019.

^§^Biting rate = number of mosquitoes collected/night/collector.

### Identification of malaria parasites

**Figure 3:**
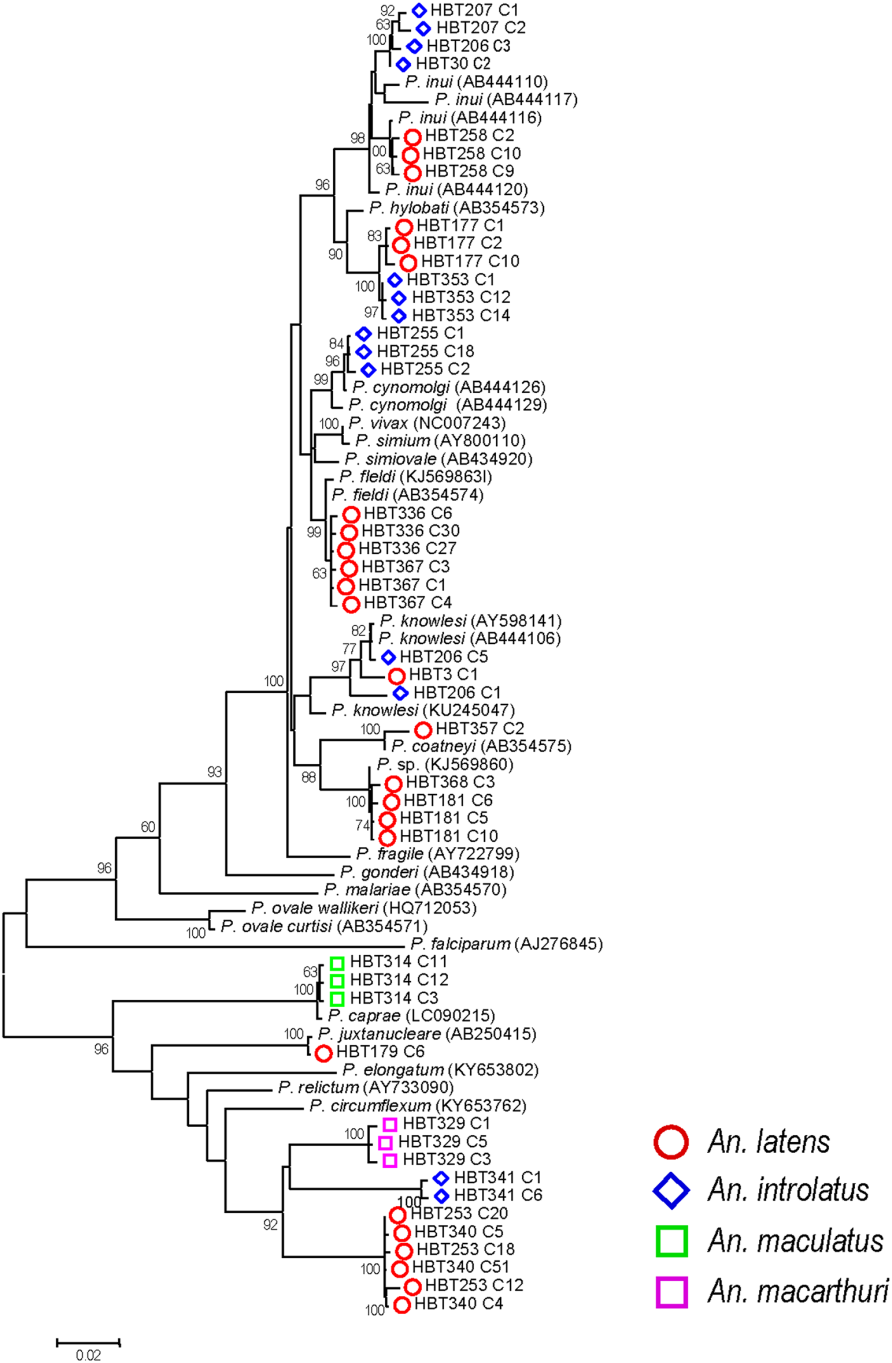
Maximum likelihood tree of *Plasmodium cox1* and its flanking sequences from the salivary glands of *Anopheles*. GenBank accession nos. of known species are in parentheses. Bootstrap values > 60% are shown along the branches. Scale indicates number of nucleotide substitutions per site. Symbols represent species of infected *Anopheles* mosquitoes.

Of 367 *Anopheles* mosquitoes analyzed, primary PCR targeting the mitochondrial cytochrome *c* oxidase subunit 1 (*cox1*) gene of *Plasmodium* revealed positive results in 19 samples, accounting for 5.2% of total samples. Most *Plasmodium*-positive salivary glands (14 of 19 samples, 73.7%) were obtained during the rainy seasons. Species-specific PCR targeting human and simian *Plasmodium* species could detect 11 mosquitoes bearing simian malarial DNA in their salivary glands including *P. inui* (n = 5), *P. fieldi* (n = 2), *P. knowlesi* (n = 1), *P. coatneyi* (n = 1), *P. cynomolgi* (n = 1) and co-existence of *P. knowlesi* and *P. inui* (n = 1). DNA sequencing of recombinant plasmid clones from *Plasmodium*-positive specimens has reaffirmed the species of almost all simian malaria parasites except two *P. inui*-positive samples (HBT177 and HBT353) whose sequences virtually belonged to *P. hylobati* (Table 2). The species of *Plasmodium* in the remaining eight mosquitoes could not be determined by species-specific nested PCR. Sequences from recombinant plasmid clones of these unassigned samples displayed six different *Plasmodium* species/lineages by phylogenetic analysis. Of these, two mosquitoes (HBT181 and HBT368) harbored *Plasmodium* species that had phylogenetic affinity to an unnamed simian malaria parasite known to infect long-tailed and pig-tailed macaques in Malaysian Borneo (GenBank accession no. KJ569860) (Fig. 3)^34^. One sample yielded the *cox1* sequence belonging to *P. juxtanucleare* with 99.79% sequence identity. Four other mosquitoes (HBT253, HBT329, HBT340 and HBT341), each containing 2 to 3 distinct *cox1* alleles, seemed to diverge from *P. circumflexum* and were placed into three distinct phylogenetic clades. The *p*-distance (*d* ± S.E.) between the *cox1* and its flanking sequences of *P. circumflexum* and those of the three distinct clades varied from 0.0577 ± 0.0057 to 0.0678 ± 0.0064 which were greater than those between some other known primate *Plasmodium* species (e.g. *P. vivax* vs. *P. simiovale*, *d* ± S.E. = 0.0139 ± 0.0028, and *P. inui* vs*. P. hylobati d* ± S.E. = 0.0237 ± 0.0039 to 0.0307 ± 0.0043). Interestingly, the remaining isolate (HBT314) contained three distinct alleles and displayed 99.71% to 99.85% sequence identity with that of *P. caprae* (GenBank accession no. LC090215). It is noteworthy that the majority of *Plasmodium* in the mosquito salivary glands contained clonal variation in the *cox1*and its flanking sequences except isolates HBT30, HBT368 and HBT179 in which single clones of *P. knowlesi, P.* sp. (KJ569860) and *P. juxtanucleare*, respectively, could be obtained.

**Table 2:** Simian and other malaria parasites in anopheline mosquitoes.

Sample ID	*Anopheles* species	Season, year	Collection time	*Plasmodium* detection method	
				Species-specific PCR	Sequencing^#^
HBT3	*An. latens*	Rainy, 2018	19.00–20.00	*P. knowlesi*	*P. knowlesi*
HBT30	*An. introlatus*	Rainy, 2018	20.00–21.00	*P. inui*	*P. inui*
HBT177	*An. latens*	Dry, 2019	19.00–20.00	*P. inui*	*P. hylobati*
HBT179	*An. latens*	Dry, 2019	19.00–20.00	Unknown	*P. juxtanucleare*
HBT181	*An. latens*	Dry, 2019	20.00–21.00	Unknown	*P.* sp. (KJ569860)
HBT206	*An. introlatus*	Dry, 2019	21.00–22.00	*P. knowlesi* + *P. inui*	*P. knowlesi* + *P. inui*
HBT207	*An. introlatus*	Dry, 2019	20.00–21.00	*P. inui*	*P. inui*
HBT253	*An. latens*	Rainy, 2019	18.00–19.00	Unknown	*P.* sp. (novel A)
HBT255	*An. introlatus*	Rainy, 2019	20.00–21.00	*P. cynomolgi*	*P. cynomolgi*
HBT258	*An. latens*	Rainy, 2019	19.00–20.00	*P. inui*	*P. inui*
HBT314	*An. maculatus*	Rainy, 2019	23.00–24.00	Unknown	*P. caprae*
HBT329	*An. macarthuri*	Rainy, 2019	20.00–21.00	Unknown	*P.* sp. (novel B)
HBT336	*An. latens*	Rainy, 2019	20.00–21.00	*P. fieldi*	*P. fieldi*
HBT340	*An. latens*	Rainy, 2019	18.00–19.00	Unknown	*P.* sp. (novel A)
HBT341	*An. introlatus*	Rainy, 2019	18.00–19.00	Unknown	*P.* sp. (novel C)
HBT353	*An. introlatus*	Rainy, 2019	18.00–19.00	*P. inui*	*P. hylobati*
HBT357	*An. latens*	Rainy, 2019	18.00–19.00	*P. coatneyi*	*P. coatneyi*
HBT367	*An. latens*	Rainy, 2019	19.00–20.00	*P. fieldi*	*P. fieldi*
HBT368	*An. latens*	Rainy, 2019	18.00–19.00	Unknown	*P.* sp. (KJ569860)

^#^Sequencing of recombinant plasmid clones.

### Diversity of simian *Plasmodium* species from mosquitoes, humans and macaques in Thailand

**Figure 4:**
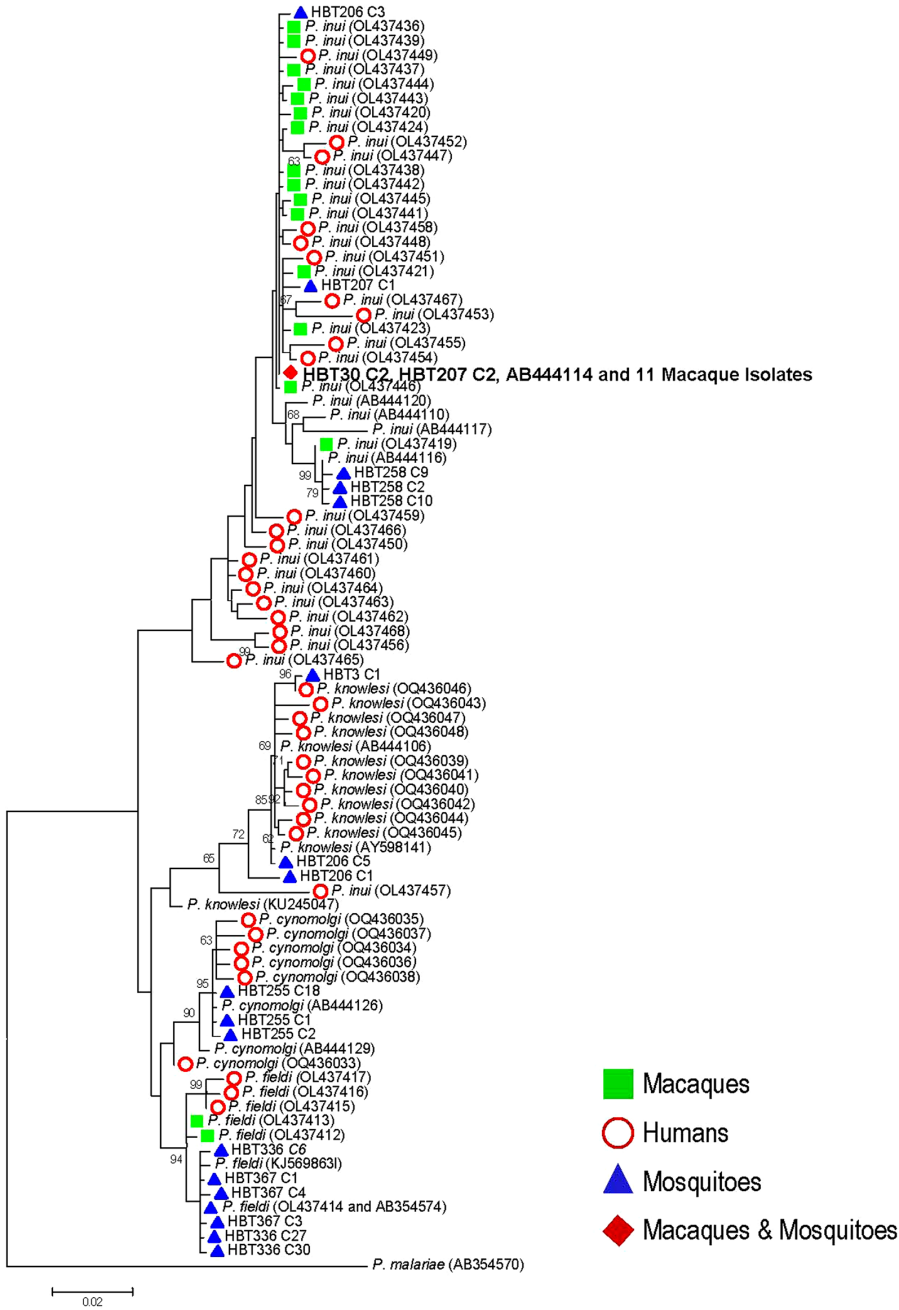
Maximum likelihood tree inferred from the *cox1* and its flanking sequences of *Plasmodium knowlesi*, *P. cynomolgi*, *P. inui* and *P. fieldi* from mosquitoes, macaques and humans in Thailand^12,13^. GenBank accession nos. are in parentheses. Bootstrap values > 60% are shown along the branches. Scale indicates number of nucleotide substitutions per site. Symbols represent host origins of taxons.

To determine allelic variation in the *cox1* and its flanking sequences of simian malaria parasites among different hosts, the sequences obtained from the salivary glands of mosquitoes in this study were compared with the corresponding gene sequences previously reported from macaques and humans in Thailand^12,13^. Results revealed that the *cox1* and its flanking sequences of *P. knowlesi*, *P. cynomolgi* and *P. fieldi* were different among isolates from mosquitoes, macaques and humans. Although allelic variation in this locus occurred among *P. inui* isolates, the sequences from mosquitoes HBT30 and HBT207 were identical with 11 macaque isolates from Cho-airong, Waeng and Sukhirin Districts in Narathiwat Province and Kabang District in Yala Province collected during 2008–2018^12,13^. Furthermore, these sequences were shared with that of *P. inui* (AB444114) strain IC (Leucosphyrus)(ATCC 30,195) isolated from *Anopheles leucosphyrus* from Negri Semilan in Malaysia since 1964 (Fig. 4A)^35^.

### Molecular identification of Anopheles species

**Figure 5:**
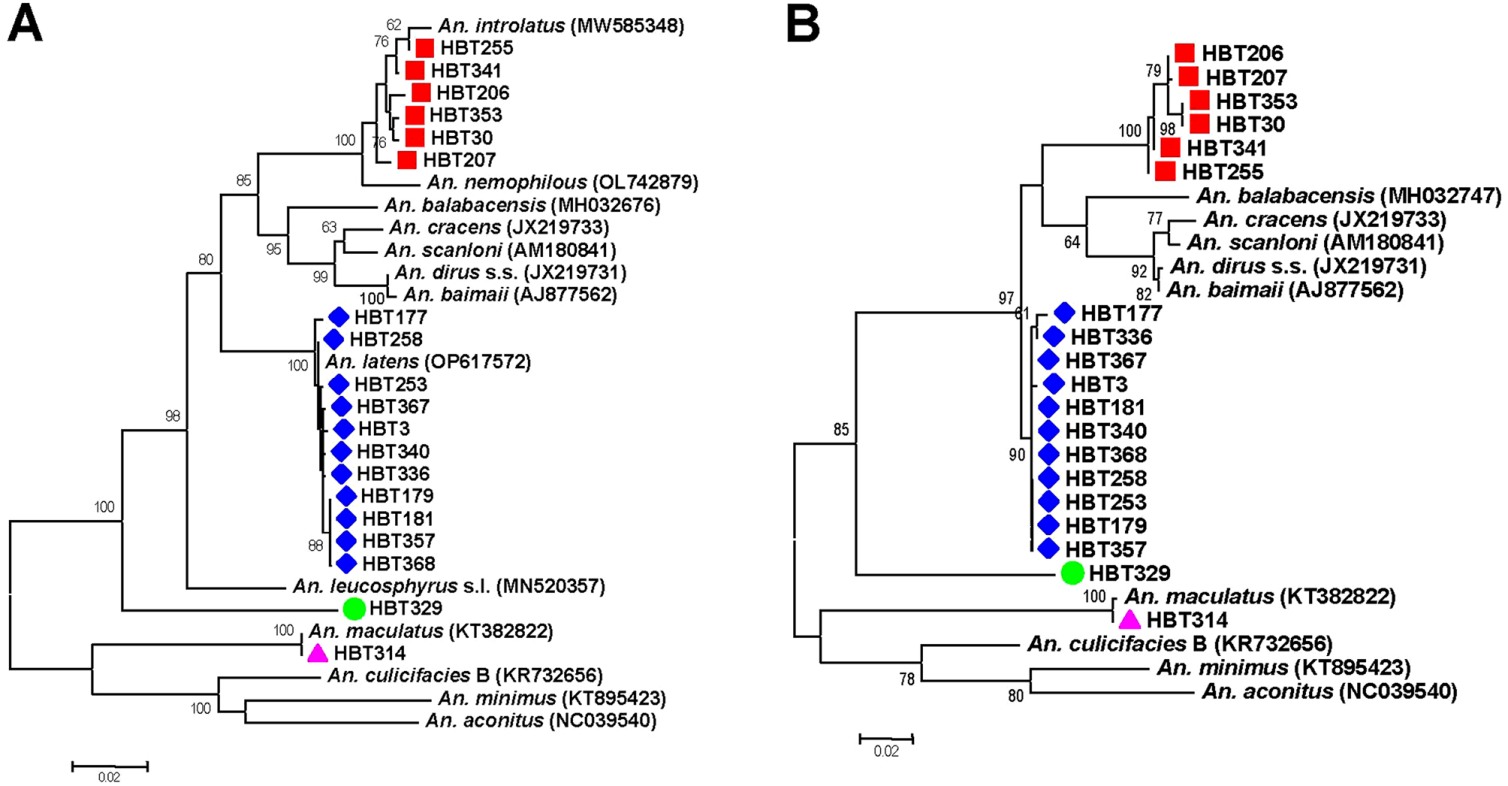
Maximum likelihood tree constructed from the *Anopheles cox1* (**A**) and *cox2* (**B**) loci. GenBank accession nos. are in parentheses. Each symbol represents identical species. Bootstrap values > 60% are shown along the branches. Scale indicates number of nucleotide substitutions per site.

**Figure 6:**
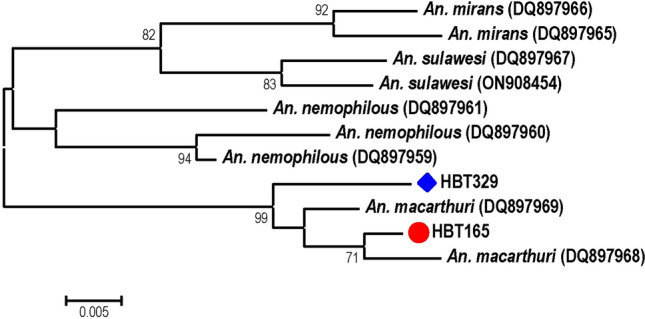
Maximum likelihood tree created from *Anopheles cox1* sequences spanning 250 bp. GenBank accession nos. are in parentheses. Bootstrap values > 60% are indicated along the branches. Scale represents number of nucleotide substitutions per site. *Plasmodium*-infected and -uninfected *Anopheles* mosquitoes are marked by blue diamond and red spot, respectively.

The species of all *Plasmodium*-positive mosquitoes were determined by sequencing of the PCR-amplified 2140 bp region of *Anopheles* mitochondrial genes encompassing *cox1* and *cox2.* Due to the lack of representative *cox2* sequences of some members in the Leucosphyrus Group, species assignment was determined mainly from the *cox1* phylogeny (Fig. 5A). Results revealed that *An. introlatus* carried 4 species of primate malaria parasites including *P. knowlesi*, *P. cynomolgi*, *P. inui* and *P. hylobati*, and a plausible novel species (HBT341) of an avian malaria parasite related to *P. circumflexum*. In the salivary glands of *An. latens*, six species of primate malaria parasites were identified including *P. knowlesi, P. inui, P. fieldi, P. hylobati*, *P. coatneyi* and an unnamed macaque malaria parasite *Plasmodium* sp. (GenBank accession no. KJ569860). Furthermore, *P. juxtanucleare* and a plausible novel species of avian plasmodia were also found in *An. latens* (Table 2). It is noteworthy that *P. caprae* was detected in the salivary glands of *An. maculatus* in this study. Although the sequence spanning *cox1* and *cox2* of mosquito HBT329 bearing an unknown *Plasmodium* sp. related to *P. circumflexum* could not be assigned from BOLD database^36^, it has been identified as *An. macarthuri* from phylogenetic analysis inferred from available reference *cox1* sequences of Leucosphyrus mosquitoes spanning the 250-bp fragments (Fig. 6). Likewise, the other unassigned species of mosquito (HBT165) without *Plasmodium* infection also belonged to *An. macarthuri* based on phylogenetic analysis (Fig. 6). Meanwhile, the tree constructed from the *cox2* locus per se displayed concordant phylogenetic affinity of mosquitoes with that inferred from the *cox1* locus (Fig. 5B).

### Feeding time of potential vector species

Both *Plasmodium*-infected *An. latens* and *An. introlatus* were captured during 18:00 and 21.00 h. Likewise, *An. macarthuri* (HBT329) bearing a plausible novel avian malaria parasite was caught between 20:00 and 21:00 h. Although most *An. maculatus* in these surveys had maximum feeding time between 21:00 and 23:00 h (Fig. 2B), the infected mosquito was caught during 23:00 and 24:00 h (Table 2).

## Discussion

In Thailand, the past couple of decades have seen a dramatic decline in the number of falciparum malaria patients while a relative increase in the proportion of vivax malaria has been envisaged, suggesting that currently applied control measures seem to be less effective against non-falciparum infections^10–13,37,38^. Likewise, an increased prevalence of patients infected with *P. knowlesi* has been observed during the past decade, especially among those who resided in areas where infected domesticated or wild macaques co-existed^10–13,37^. In 2022, a total of 10,152 malaria cases were reported in Thailand of which *P. vivax*, *P. falciparum* and *P. knowlesi* were incriminated in 95.3%, 2.5% and 1.8% of infections, respectively^37^. Importantly, control of zoonotic transmission of simian malaria in humans would require integrative knowledge about the prevalence of simian malaria parasites in humans, macaque natural hosts and *Anopheles* vectors. Our surveys in Narathiwat Province have revealed a relatively high prevalence of *Anopheles* mosquitoes carrying malaria parasites in their salivary glands (5.16%, 19 of 368 mosquitoes) in which 13 of these belonged to primate *Plasmodium* species. This is in stark contrast with the absence of infected mosquitoes in a recent survey in Surat Thani Province, another region in Southern Thailand^39^. Differential risk of acquiring malaria in different endemic areas could stem from variation in bionomics of mosquito vectors, prevalence of malaria patients and status of reservoir hosts. In this study we deployed both PCR and sequencing to detect primate malarial DNA in the mosquitoes’ salivary gland samples which revealed concordant results in most samples. Discrepancy between PCR and sequencing in samples HBT177 and HBT353 (Table 2) could stem from cross hybridization of the nested PCR primers targeting the *cox1* sequence of *P. inui* and a variant of *P. hylobati* identified in this study which possessed different sequences of the primer regions from that of *P. hylobati* strain WAK isolated from a gibbon (*Hylobates molock*) in Sarawak (GenBank accession no. AB354573)^40^. However, differentiation between *P. inui* and *P. hylobati* could be resolved and correctly assigned by analysis of the *cox1* sequences as shown in our previous study^13^. It is noteworthy that *P. hylobati* has not been detected among macaques in Thailand^13,31,32^ while the prevalence of this simian malaria species in gibbons known as its natural hosts awaits further investigation.

The presence of *P. knowlesi* DNA in the salivary glands of *An. latens* and *An. introlatus* in Narathiwat Province has implied that both mosquito members in the Leucosphyrus complex could serve as potential vectors of this simian malaria species in this province where both infected humans and macaques have been previously identified^10–13,31,32^. Mosquitoes belonging to the Leucosphyrus Subgroup comprise  at least 13 species while seven of these have been detected in Thailand^33^. The breeding places of these mosquitoes included ground pools, animal footprints, wheel-tracks and small shallow running streams in the forested ecotype with partial or heavily-shaded areas^33,41^. While *An. introlatus* seemed to occupy Peninsular Malaysia and Southern Thailand, previous surveys have documented that *An. latens* was widely distributed across at least 3 countries in Southeast Asia including Kalimantan of Indonesia, Malaysian Borneo, western regions of Peninsular Malaysia and Southern Thailand encompassing Chumporn, Nakhon Si Thammarat, Phang Nga, Satun, Songkhla and Narathiwat Provinces^33,41^. Consistently, *An. latens* has been found to vector *P. knowlesi* in Kapit, Sarawak^19^. Both *An. introlatus* and *An. hackeri* have been incriminated in transmission of this simian malaria species in Selangor, Peninsular Malaysia^21,42^. Other members in the Leucosphyrus Group as potential vectors of knowlesi malaria include *An. dirus* in Vietnam^27,28^, *An. cracens* in Pahang^18^ and *An. balabacensis* in Sabah and Sarawak in Malaysian Borneo and Palawan in The Philippines^22–26^. It is noteworthy that transmission of knowlesi malaria could be from other groups of mosquitoes including *An. donaldi* belonging to the Barbirostris Group in Sabah and Sarawak^23^, *An. sundaicus* species D in Andaman and Nicobar Islands^30,43^ and two members in the Umbrosus Group, *An. collessi* and *An. roperi*, in Sarawak^25,29^. The occurrence of diverse species of *Anopheles* potential vectors of *P. knowlesi* could enhance transmission of this simian malaria parasite in various ecological niches.

Besides being vectors of *P. knowlesi* in Thailand, it is plausible that *An. latens* could be a natural vector of *P. inui* and *P. fieldi*. Likewise, *An. introlatus* seemed to be responsible for transmission of *P. cynomolgi* and *P. inui* in the southern part of the country. Our findings have supported previous studies that both *An. latens* and *An. introlatus* could be important vectors for simian malaria transmissible to humans^19–21^. Furthermore, *An. latens* could serve as a vector of *P. coatneyi* while the presence of *P. coatneyi* and *P. hylobati* DNA in the salivary glands of *An. introlatus* has suggested that they could transmit these simian malarias in the survey areas. In this study, clonal diversity in the *cox1* sequences of most primate malaria parasites from infected mosquito salivary glands (Figs. 3, 4) and co-existence of different species of *Plasmodium* in a single mosquito (*An. introlatus* sample HBT206, Table 1) could probably stem from multiple feedings of the vectors on different monkeys infected with variant strains or different species of *Plasmodium.* Alternatively, multiple clones/species of malaria parasites in individual macaques could be prevalent, lending plausibility for the mosquitoes to acquire multiple clones/species of plasmodia upon single feedings. Our previous study has shown considerable clonal diversity of *P. knowlesi* and other primate malaria parasites in both long-tailed and pig-tailed macaques in Southern Thailand^32^; thus, the latter scenario could not be excluded. Within mosquitoes, inter-species and intra-species competition could further influence subsequent transmission of malaria parasites to the vertebrate hosts^44,45^. Meanwhile, multiclonal and mixed species infections with simian malaria in humans occurred less frequently than in mosquitoes and macaques in Thailand^12,13,31,32^. Intriguingly, differential capability of adaptation to humans might occur among strains/lineages of primate *Plasmodium* species while genetic/phenotypic diversity of these parasites could contribute to a range of disease severity as observed in knowlesi malaria in humans albeit multiple host factors may also play crucial roles^4,5,8,10–13,46,47^.

It has been shown that fluctuation in the levels of asexual parasitemia and gametocytemia occurred in experimental *P. knowlesi* infections in long-tailed macaques with peak parasitemia between 18:00 and 24:00 h and between 24:00 and 06:00 h, respectively^48^. The biting period of *An. latens* in Sarawak during 19:00 and 06:00 h seemed to coincide with the parasitemia of both asexual and sexual stages in peripheral blood of infected hosts; thus, enhancing transmission of simian malaria^16,19,23^. In field studies, *An. latens* and *An. introlatus* had peak biting activity between 19:00 and 20:00 h and 20:00 and 21:00 h, respectively^16,20,43^. Consistently, in this study simian *Plasmodium*-infected *An. latens* and *An. introlatus* were captured between 18:00 and 21:00 h (Table 2), implying that exposure to mosquito bites in areas where infected macaques coexist in Southern Thailand during the first quarter of the night may pose a high risk for getting zoonotic malaria.

Phylogenetic trees inferred from the *Plasmodium cox1* sequences derived from humans, macaques and *Anopheles* mosquitoes in Thailand have revealed genetic diversity across these hosts (Fig. 4). Despite limited number of available sequences, it is noteworthy that human isolates of *P. knowlesi*, *P. cynomolgi*, *P. inui* and *P. fieldi * differed from those derived from macaques and *Anopheles* mosquitoes, implying that the extent of genetic variation in natural populations of these primate malaria parasites could be more extensive. However, it seemed that a certain strain of *P. inui* derived from *An. latens* and *An. introlatus* in this study shared identical *cox1* and its flanking sequences across 1504 bp with 11 previously characterized isolates from pig-tailed macaques from Cho-airong, Waeng and Sukhirin Districts in Narathiwat Province obtained in 2008, 2013, 2018 and 2020^13,32^. These isolates also possessed the same sequence as that of strain IC (*leucosphyrus*) derived from *An. leucosphyrus* caught from Negri Semilan in Malaysia in 1964^49^. Although identical mitochondrial *cox1* sequences may not represent the same entire nuclear genomes, there remains a possibility that certain strains of *P. inui* could predominate and persist in natural transmission cycle that could probably stem from their adaptive capabilities to survive in diverse species of mosquito vectors and reservoir hosts.

Our sequence analysis of recombinant clones from 8 infected mosquitoes whose species of malaria parasites could not be assigned by PCR has revealed that 2 samples (HBT181 and HBT368) belonged to an unnamed *Plasmodium* known to infect both pig-tailed and long-tailed macaques in Sabah, northern Borneo (GenBank accession no. KJ569860)^34^. The placement of these isolates in the phylogenetic tree seemed to be closely related to *P. coatneyi* while the magnitude of evolutionary distance between the *cox1* and its flanking sequences of these malaria species and that of *P. coatneyi* was comparable or greater than those between other primate *Plasmodium* species. Therefore, both isolates from *An. latens* (HBT181 and HBT368) and the previously described isolates (KJ569860) from Sabah^34^ could belong to a distinct species. It is likely that more novel species of primate malaria parasites remain to be discovered. Meanwhile, *Plasmodium* species from *An. maculatus* (HBT314) displayed 99.77% to 99.92% sequence identity with that of *P. caprae* (GenBank accession no. LC090215). This ungulate malaria parasite has caused infections in domestic goats in Western Thailand and other countries^50^. It is plausible that *An. maculatus* could serve as a vector of *P. caprae* in the southern part of Thailand akin to *An. subpictus* and *An. aconitus* that have been recently reported to be implicated in transmission of *P. caprae* among goat farms in the western provinces of the country^51^.

Apart from primate malaria, our analysis has identified *An. latens* as a potential vector of *P. juxtanucleare*, a predominant avian malaria parasite that infects chicken (*Gallus gallus domesticus*) and fighting cocks (Burmese red junglefowl, *Gallus gallus spadiceus*) in Thailand. Meanwhile, this avian malaria parasite has been known to infect other members of the Phasianidae^52–54^ including short-crested flycatcher in Brazil^55^, turkey, parrot and chugar in Pakistan^56^, eared-pheasant in Japan^57^ and black-footed penguins in South Africa^58^. Besides cosmopolitan in distribution, *P. juxtanucleare* has been detected among wild passerines in Brazil, suggesting spillover from poultry to free-living birds^59^. Although *P. juxtanucleare* is known to be mainly vectored by mosquitoes in the genus *Culex* (e.g. *Cx. vishnui* and *Cx quinquefasciatus* in The Philippines and *Cx. saltanensis* in Brazil)^60,61^, identification of *An. latens* as an additional vector in our study has expanded a range of mosquitoes capable of transmitting this avian malaria species. Herein, we also identified 3 *Plasmodium* lineages from *An. latens* and *An. introlatus* whose *cox1* sequences displayed phylogenetic clustering within avian *Plasmodium* clades. Furthermore, the evolutionary distance (*p*-distance) between these lineages and a closely related *P. circumflexum* was comparable to or greater than those between known primate and avian malarial species, suggesting that these newly identified lineages could represent distinct avian *Plasmodium* species. Taken together, it is plausible that a wide range of mosquito vectors and diverse avian host species could enhance cosmopolitan distribution of avian malaria parasites.

Despite a wealth of knowledge about morphological differences among *Anopheles* vectors of malaria, molecular identification remains to be a useful method for fine resolution of mosquito species, especially those with minor structural differences. Although our sequence analysis encompassing near complete *cox1* and partial *cox2* sequences, *An. latens* and *An. introlatus* could be identified based on available *cox1* reference sequences in the public database. The concordant topologies of phylogenetic trees inferred from either *cox1* or *cox2* sequences have provided an additional marker for speciation of these mosquito species (Fig. 5).

The midguts and ovaries of the mosquitoes have not been examined in this study. Although the presence of malarial DNA in the midguts may not replicate the results obtained from the mosquito’s salivary gland samples, it can support the integrity of analysis from salivary gland specimens. Likewise, more information on the bionomics of the mosquitoes from examination of the ovary samples has not been available in this study. It is noteworthy that the presence of malarial DNA or sporozoites in the mosquito’s salivary glands does not directly indicate its true vectorial status^1^. However, consistent identification of specific malarial DNA from the same species of mosquitoes, especially those from different geographic areas, seems to indicate that they could vector a given *Plasmodium* species^16^.

To the best of our knowledge, this is the first report on *An. latens* and *An. introlatus* as vectors of *P. knowlesi* in Thailand while both species could potentially transmit other simian and some avian plasmodia. Furthermore, *An. maculatus* has been identified to vector *P. caprae* that may parasitize domestic goats in the southern part of the country. Unraveling vectors of malaria and their bionomics could contribute to knowledge about disease transmission in malaria endemic areas that are important for prevention and control policy.

## Materials and methods

### Study area

Mosquito collections were conducted from three locations in Waeng (A: 5° 48′ 27″ N, 101° 50′ 44″ E; B: 5° 49′ 12″ N, 101° 51′ 20″ N and C: 5° 50′ 5″ N, 101° 51′ 7″ E) and three sites in Sukhirin Districts (D: 5° 48′ 4″ N, 101° 45′ 2″ E; E: 5° 48′ 8″ N, 101° 45′ 13″ N and F: 5° 47′ 40″ N, 101° 42′ 51″ E) in Narathiwat Province during 2018 and 2019 (Fig. 1). These locations were selected because (i) indigenous villagers residing in these areas were diagnosed to be infected with *P. knowlesi*^10–13^, (ii) both wild and domesticated pig-tailed and long-tailed macaques were prevalent and known to be infected with several simian *Plasmodium* species^32^, (iii) both districts were situated along forest fringe with natural streams and rivers running through the areas that could be natural breeding places for *Anopheles* mosquitoes and (iv) rubber plantations were located in the districts where farmers and workers were frequently exposed to mosquito bites during the harvesting process. The collection sites had a tropical monsoon climate and were located near Hala-Bala tropical rain forest, covering an area of approximately 1.3 square kilometers across Yala and Narathiwat Provinces along Thailand-Malaysia border approximately between 5° 37′–6° 14′ N and 101° 8′ E–101° 51′ E. The elevation of Hala-Bala Forest ranged from 50 m to 1.5 km above mean sea level with annual mean temperature of 27.6 °C, annual average rainfall of 2560 mm, and relative humidity between 77 and 80%. The heavy rainy months of the year were from November to December and the relatively dry season from February to April. The number of days with rainfall (≥ 1.0 mm) occurred approximately 200 days per annum.

### Collection of mosquitoes

Mosquito collections were carried out every other night for 10 days during each of the four surveys in March and August 2018 and March and November 2019, covering relatively dry and rainy seasons of the study sites. Human-landing catches of mosquitoes were done outdoors. Each collection site comprised two teams, each consisting of two persons: one volunteer served as bait and the other as collector. The first team captured the mosquitoes from 18:00 to 24.00 h and the other from midnight until dawn (06:00 h). The collector captured the mosquitoes upon landing on the human bait by using a plastic collection tube. All captured mosquitoes were kept individually in a 1.5 ml sterile microtube with cap, placed on ice and transferred to 4 °C refrigerator for subsequent morphological identification. All volunteers and field staff were monitored for malaria infection during the field work and thereafter on a weekly basis for 2 months.

### Morphological identification of mosquitoes and dissection

Each mosquito was examined under stereomicroscope to exclude those belonging to genera other than *Anopheles*. All female *Anopheles* mosquitoes were gently fixed onto plasticine using ultra-thin micro-headless pins. Morphological identification was based on characteristic features of *Anopheles* genera, groups, complex and species according to illustrated keys to the mosquitoes of Thailand^33^. After morphological identification, the salivary glands of each female *Anopheles* were dissected and preserved in absolute ethanol in separate tubes per individual mosquitoes.

### Preparation of DNA

Ethanol was allowed to evaporate from mosquito’s salivary glands prior to DNA preparation. Extraction and purification of DNA were performed for each salivary gland sample by using DNeasy Blood and Tissue Kit following the manufacturer’s protocol (Qiagen, Hilden, Germany). DNA was kept at − 40 °C until use.

### Molecular detection of Plasmodium species

DNA extracted from salivary glands of each *Anopheles* mosquito was used as template for species-specific PCR detection targeting the mitochondrial cytochrome *c* oxidase subunit 1 (*cox1*) gene of human and simian malaria parasites including *P. falciparum*, *P. vivax*, *P. malariae, P. ovale*, *P. knowlesi*, *P. cynomolgi*, *P. inui*, *P. coatneyi* and *P. fieldi*. Amplification reaction mixtures and PCR conditions were the same as those previously described^12,13^. The primary PCR products of all *Plasmodium*-positive samples were subcloned into plasmid pGEM-T Easy Vector Systems (Promega, Madison, Wisconsin, USA) with *Escherichia coli* strain JM109 as a host for transformation. At least five recombinant clones from each positive sample were used as templates for determination of the *cox1* sequences.

### Molecular detection of Anopheles species

Primers for PCR amplifications of *Anopheles* mosquitoes’ DNA were derived from the mitochondrial genes encompassing cytochrome *c* oxidase subunits I and II intervened by the gene encoding tRNA-Leu. The forward and reverse primers were ALMTF0 (5ʹ-ATTTAATCGCGACAATGATTATTTTC-3ʹ) and ALMTR0 (5ʹ-CTATGATTTGCTCCACAAATTTC-3ʹ), respectively. Amplification of the *Anopheles* DNA was performed in a total volume of 30 μl of the reaction mixture containing template DNA, 300 mM each deoxyribonucleoside triphosphate, 3 μl of 10X LA Taq PCR buffer, 2.5 mM MgCl_2_, 0.3 μM of each primer and 1.25 units of LA Taq DNA polymerase (Takara, Seta, Japan). Amplification profile consisted of preamplification denaturation at 94 °C for 1 min followed by 35 cycles of 96 °C for 20 s, 55 °C for 30 s and 72 °C for 4 min, and a final extension at 72 °C for 10 min. Thermal cycling of the samples was performed in a GeneAmp 9700 PCR thermal cycler (Applied Biosystems, Foster City, CA). The amplicons were examined by 1% agarose gel electrophoresis and visualized under a UV transilluminator. The PCR product was purified by using QIAquick PCR purification kit (Qiagen, Germany) prior to DNA sequencing. Sequencing primers were used for obtaining overlapping DNA sequences. Sequencing was performed on an ABI3100 Genetic Analyzer using the BigDye Terminator v3.1 Cycle Sequencing Kit (Applied Biosystems, USA). Mosquitoes belonging to Leucosphysrus Group were further identified by using multiplex PCR assay for differentiation of *An. latens*, *An. introlatus*, *An. cracens* and *An. balabacensis* as reported^62^.

### Data analysis

Nucleotide sequences were aligned by using the codon-based option in MUSCLE program with manual adjustment^63^. For *cox1* sequences of malaria parasites, known species or lineage of *Plasmodium* were used for comparative analysis which included the following GenBank accession nos.: *P. falciparum* (AJ276845), *P. vivax* (NC007243), *P. malariae* (AB354570), *P. ovale curtisi* (AB354571), *P. ovale wallikeri* (HQ712053), *P. knowlesi* (AB444106 and AY598141), *P. cynomolgi* (AB434919 and AB444126), *P. inui* (AB444110, AB444111, AB444114, AB444116 and AB444120), *P. fieldi* (AB354574 and KJ5698631), *P. coatneyi* (AB354575), *P. fragile* (AB444135 and AY722799), *P. hylobati* (AB354573), *P. gonderi* (AB434918), *P. simium* (AY800110), *P. simiovale* (AB434920), *P. circumflexum* (KY653762), *P. juxtanucleare* (AB250415), *P. relictum* (AY733090), *P. elongatum* (KY653802), *P. caprae* (LC090215) and *P.* sp. (KJ569860). The *cox1* sequences of human- and macaque-derived *P. knowlesi*, *P. cynomolgi*, *P. inui* and *P. fieldi* in Thailand from our previous studies were included for comparison (GenBank accession nos. OL437412–OL437468 and OQ436033–OQ436048)^12,13^. For *Anopheles cox1* and *cox2* references, both complete and partial sequences of known species were deployed in the phylogenetic analysis: *An. introlatus* (MW585348), *An. nemophilous* (DQ897959–DQ897961 and OL742879), *An. balabacensis* (MH032676 and MH326747), *An. cracens* (JX219733, *An. scanloni* (AM180841), *An. dirus* s.s (JX219731), *An. baimaii* (AJ877562), *An. leucosphyrus* s.l. (MN520357), *An. latens* (OP617572), *An. maculatus* (KT382822), *An. minimus* (KT895423), *An. culicifacies* B (KR732656), *An. aconitus* (NC039540), *An. mirans* (DQ897965 and DQ897966), *An. sulawesi* (DQ890954 and ON908454) and *An. macarthuri* (DQ897968 and DQ897969). All sites at which the alignment postulated a gap were eliminated in pairwise comparisons of the analysis. For evolutionary distance between sequences, *p*-distance was determined by dividing the number of nucleotide differences between two sequences by the total number of nucleotides compared and its standard error (*d* ± S.E.) between sequences was computed by 1000 bootstrap pseudoreplicates. Phylogenetic trees were constructed by using the maximum likelihood method with the best substitution model for the sequence data that yielded the minimum Bayesian Information Criterion (BIC) scores^64^. Confidence levels of clustering patterns in the phylogenetic tree were assessed by 1000 bootstrap pseudoreplicates.

### Ethical approval

This study was reviewed and approved by the Institutional Review Board in Human Research of Faculty of Medicine, Chulalongkorn University (IRB No. 272/61, COA No. 841/2019) and Naresuan University Institutional Review Board of Human Research (IRB No. 0614/62, COA No. 057/2020). Prior to serving as human baits for mosquito collection, written informed consent was obtained from all participants. All procedures were performed in accordance with the relevant guidelines and regulations.

## Data Availability

The datasets generated during and/or analyses during the current study are available from the corresponding authors upon request.

## Code availability

Forty-five sequences of the mitochondrial *cox1*and its flanking sequences of *Plasmodium* and 19 sequences of *Anopheles* mitochondrial genes spanning *cox1* and *cox2* have been deposited in NCBI GenBank under accession numbers OQ473193–OQ473237 and OQ466059–OQ466077, respectively.
